# *Toxoplasma gondii* Impairs Myogenesis *in vitro*, With Changes in Myogenic Regulatory Factors, Altered Host Cell Proliferation and Secretory Profile

**DOI:** 10.3389/fcimb.2019.00395

**Published:** 2019-11-27

**Authors:** Paloma de Carvalho Vieira, Mariana Caldas Waghabi, Daniela Gois Beghini, Danilo Predes, Jose Garcia Abreu, Vincent Mouly, Gillian Butler-Browne, Helene Santos Barbosa, Daniel Adesse

**Affiliations:** ^1^Laboratório de Biologia Estrutural, Instituto Oswaldo Cruz, Fiocruz, Rio de Janeiro, Brazil; ^2^Laboratório de Genômica Funcional e Bioinformática, Instituto Oswaldo Cruz, Fiocruz, Rio de Janeiro, Brazil; ^3^Laboratório de Inovação em Terapias, Ensino e Bioprodutos, Instituto Oswaldo Cruz, Fiocruz, Rio de Janeiro, Brazil; ^4^Laboratório de Embriologia de Vertebrados, Instituto de Ciências Biomédicas, Universidade Federal do Rio de Janeiro, Rio de Janeiro, Brazil; ^5^Sorbonne Université, INSERM, Institut de Myologie, Myology Research Center UMRS974, Paris, France

**Keywords:** *Toxoplasma gondii*, myogenesis, C2C12 cells, myotube, myogenic regulatory factor, congenital toxoplasmosis

## Abstract

*Toxoplasma gondii* is the causative agent of toxoplasmosis, a parasitic disease with a wide global prevalence. The parasite forms cysts in skeletal muscle cells and neurons, although no evident association with inflammatory infiltrates has been typically found. We studied the impact of *T. gondii* infection on the myogenic program of mouse skeletal muscle cells (SkMC). The C2C12 murine myoblast cell line was infected with *T. gondii* tachyzoites (ME49 strain) for 24 h followed by myogenic differentiation induction. *T. gondii* infection caused a general decrease in myotube differentiation, fusion and maturation, along with decreased expression of *myosin heavy chain*. The expression of Myogenic Regulatory Factors Myf5, MyoD, Mrf4 and myogenin was modulated by the infection. Infected cultures presented increased proliferation rates, as assessed by Ki67 immunostaining, whereas neither host cell lysis nor apoptosis were significantly augmented in infected dishes. Cytokine Bead Array indicated that IL-6 and MCP-1 were highly increased in the medium from infected cultures, whereas TGF-β1 was consistently decreased. Inhibition of the IL-6 receptor or supplementation with recombinant TGF-β failed to reverse the deleterious effects caused by the infection. However, conditioned medium from infected cultures inhibited myogenesis in C2C12 cells. Activation of the Wnt/β-catenin pathway was impaired in *T. gondii*-infected cultures. Our data indicate that *T. gondii* leads SkMCs to a pro-inflammatory phenotype, leaving cells unresponsive to β-catenin activation, and inhibition of the myogenic differentiation program. Such deregulation may suggest muscle atrophy and molecular mechanisms similar to those involved in myositis observed in human patients.

## Introduction

*Toxoplasma gondii* is an obligate intracellular protozoan parasite that can cause a devastating disease in immune-compromised patients and fetuses (Montoya and Liesenfeld, 2004; Dubey, 2008). Transmission occurs by ingestion of tissue cysts, present in undercooked meat, or by ingestion/inhalation of sporulated oocysts that are shed along with the feces of infected felids (Dubey and Frenkel, 1972). The cysts rupture inside the host's digestive system and release the parasites, which rapidly infect host cells and, in a few days, spread throughout the entire organism. The ability for the parasite to cause disease is directly linked to its replication inside a parasitophorous vacuole in the cytoplasm of host cells. From this vacuole, parasites scavenge nutrients from the host cell while causing reorganization of host organelles and cytoskeletal elements, preventing host cell apoptosis and altering host gene expression to its own benefit (Saeij et al., 2007; Wu et al., 2016; Acquarone et al., 2017).

Upon the host's immunological response, intracellular tachyzoites differentiate into slow-dividing bradyzoite forms, which, in turn modify the parasitophorous vacuole membrane, transforming it into the newly formed cyst wall. *T. gondii* displays an interesting interaction with post-mitotic cells, and cysts can be found in the neurons and skeletal muscle fibers of chronically infected individuals (Dubey, 1998). Intense myositis, altered electromyograms and reduced grip strength have also been reported in immunocompetent infected humans (Montoya et al., 1997; Hassene et al., 2008; Cuomo et al., 2013), suggesting that infection impairs skeletal muscle function.

In order to better characterize the interplay between *T. gondii* and skeletal muscle cells (SkMC), our group used a primary mouse SkMC culture that promotes high rates of spontaneous tachyzoite-bradyzoite conversion (Guimarães et al., 2008; Ferreira-da-Silva Mda et al., 2009) and leads to the production of inflammatory intermediates, such as prostaglandins, IFN-γ and interleukin-12 (Gomes et al., 2014). We have also described a decrease in M-cadherin content in primary SkMC cultures infected by *T. gondii* and a reduction in the number of myotubes when muscle cells were infected with the highly virulent RH strain (Gomes et al., 2011).

Myogenesis is a precisely coordinated differentiation program, starting from the first weeks of embryonic development, when somitic cells generate muscle cell progenitors, called myoblasts (Berendse et al., 2003). These elongated mononucleated cells progressively fuse to form long, multinucleated fibers called myotubes that express the differentiated gene pattern of mature muscle cells (Dedieu et al., 2002). Muscle cell early determination and differentiation are controlled by a set of transcription factors (McKarney et al., 1997), known as Myogenic Regulatory Factors (MRFs), which are active at precise developmental stages and functionally correlated to each other (De Angelis et al., 1999). Myf5 and MyoD control paraxial muscle differentiation, and both activate myogenin, known to be associated with final muscle maturation. Mrf4 plays a role in determining the fiber phenotype in postnatal life (Zhang et al., 1995), although a potential role during early development has also been suggested (Kassar-Duchossoy et al., 2004). The expression of muscle-specific proteins (such as α-actin, myosin heavy and light chain, tropomyosin, among others) is closely MRF-dependent. Myogenesis is also crucial for SkMC repair in adult life, through the activation and differentiation of adult muscle stem cells, also named satellite cells.

We investigated which mechanisms underlie myogenesis defects during *T. gondii* infection, using the C2C12 mouse myoblast cell line, since they allow for myogenic differentiation process synchronization. Using this model, we describe how *T. gondii* affects MRFs expression and other mechanisms, such as proliferation, apoptosis and cytokines/chemokines secretion and we identified defects in the Wnt/β-catenin pathway activation, which is also involved in myogenesis.

## Methods

### Cell Culture

The mouse skeletal myoblast C2C12 cell line was purchased from ATCC and maintained in a proliferation medium [PM, DMEM high glucose (Sigma Aldrich) with 10% fetal bovine serum (Cultilab, São Paulo, Brazil) and 1% antibiotic solution (Thermo Fisher)]. Before reaching confluency, cells were dissociated with Trypsin/EDTA solution in PBS and plated for experiments. For myogenesis induction, cells were cultivated in PM until reaching 70% confluency, when the medium was changed to a differentiation medium (DM, DMEM with 2% horse serum and 1% antibiotics solution).

### *T. gondii* Infection

Parasites from the ME49 strain were obtained from the brains of C57Bl/6 mice infected 45 days before isolation. Cysts were ruptured with an acid pepsin solution and free parasites were added to Vero cell (ATCC) monolayers. After 2 weeks of culture re-infections, tachyzoites released from the supernatant were collected and centrifuged prior to use. For the experiments, 60,000 C2C12 cells were plated onto 13-mm diameter glass round coverslips in 500 μl of PM per well for 24 h. Subsequently, cultures were infected with tachyzoites at a MOI of 3:1 parasite:host cell for 2 h. Cells were then washed in Ringer solution, fresh PM was added, and cells were then maintained at 37°C for an additional 22 h. After this period (total of 24 h of infection), half of the cultures were switched to DM while the other half was maintained in PM. The cultures were analyzed at 24 and 120 h after differentiation induction, corresponding to 48 and 144 h of infection, respectively.

### Immunofluorescence

Cells were plated onto 13-mm glass round coverslips in 24-well plates. At desired times, the conditioned medium was collected for cytokine analyses, as described below. Cultures were washed in PBS and fixed with 4% paraformaldehyde for 5 min at 20°C, permeabilized with a 0.5% Triton x-100 (Sigma Aldrich) solution in PBS, blocked with 4% bovine serum albumin solution for 30 min and incubated overnight with primary antibodies at 4°C. The primary antibodies used in this study and their references are listed in Table 1. Secondary antibodies goat anti-mouse conjugated to AlexaFluor 594 and donkey anti-rabbit conjugated to AlexaFluor 488 (Thermo Fisher) were incubated for 1 h at 37°C. For necrosis assessments, live cells were incubated with 40 μg/ml propidium iodide solution diluted in PBS for 10 min. As a positive control, 0.25% Triton x-100 was incubated on a separate coverslip for 5 min at 37 °C. Nuclei were visualized by incubating the cells with DAPI (4',6-diamidino-2-phenylindole dihydrochloride) at 0.2 μg/ml for 5 min at 20°C and slides were mounted in a DABCO solution containing 50% glycerol.

**Table 1:** List of primary antibodies used for the immunofluorescence assays.

**Antibody**	**Host species**	**Company name**	**Reference number**	**Dilution**
Myogenin	Mouse	DSHB	F5D-s	1:100
MyHC type II (fast twitch)	Mouse	Sigma Aldrich	M4276	1:400
MyHC type I	Mouse	DSHB	MF20	1:25
MyoD	Mouse	DSHB	D7F2-s	1:100
Desmin	Rabbit	Sigma Aldrich	D8281	1:100
Ki67	Rabbit	ABCAM	ab15580	1:80
SAG1 (P30)	Mouse	Santa Cruz Biotechnologies	Sc-52255	1:100
Cleaved Caspase-3	Rabbit	Cell signaling	9661	1:400

### Real Time qPCR

A total of 6.6 × 10^5^ cells were cultured in 60 mm plastic petri dishes (Corning) and total RNA was extracted using the RNeasy kit (Qiagen). Contamination with genomic DNA was avoided by treating the samples with DNase I (Qiagen) following the manufacturer's instructions. Concentrations were measured using a NanoDrop equipment (Thermo Fisher) and RNA samples were validated for the experiments when the 260/230 ratio was above 1.9. A total of 1 μg of total RNA was reversely transcribed into cDNA with Superscript III kit (Invitrogen). Real time PCR analyses were performed with 0.5 μL of cDNA and Power SYBR Green Master Mix (Thermo Fisher) and 0.05 μmol/L of endogenous control (PPIA) or 0.027 μmol/L of muscle-specifics primers. Cycling conditions were 94°C for 10 min, followed by 40 cycles of 94°C for 30 s and 60°C for 30 s, with a fluorescence reading at the end of each cycle. Target gene expression data were plotted as normalized by endogenous control (PPIA) and relative to uninfected cells maintained in PM for each time point, using 2^−ΔΔ*ct*^. The primer sequences used herein are listed in Table 2.

**Table 2:** List of primers used for RT-qPCR.

**Gene name**	**Sense sequence**	**Anti-sense sequence**	**References**
MyHC beta (slow twitch)	CGCAATGCAGAGTCAGTGAA	TTGCGGAACTTGGACAGGTT	Nishida et al., 2015
*myogenin*	CTACAGGCCTTGCTCAGCTC	ACGATGGACGTAAGGGAGTG	Hildyard and Wells, 2014
*MyoD*	TACAGTGGCGACTCAGATGC	GAGATGCGCTCCACTATGCT	Hildyard and Wells, 2014
*Myf5*	CTGTCTGGTCCCGAAAGAAC	AGCTGGACACGGAGCTTTTA	Hildyard and Wells, 2014
*Mrf4*	GGCTGGATCAGCAAGAGAAG	CCTGGAATGATCCGAAACAC	Hunt et al., 2013
PPIA (*Peptidyl-prolyl cis-trans isomerase*)	GGCCGATGACGAGCCC	TGTCTTTGGAACTTTGTCTGCAA	Hunt et al., 2013

### TGF-β1 Measurements

Conditioned medium was obtained from C2C12 cultures at the different experimental conditions analyzed, as described above. To obtain conditioned medium for cytokine assays, each well of 24-well plates was incubated with 300 μl of either PM or DM for 1 day. The medium was collected in 1.5 ml centrifuge tubes and kept on ice, centrifuged at 14,000 rpm for 5 min. Supernatants were then transferred to new tubes and the conditioned medium was kept at −80°C until use. Total TGF-β1 levels present in the conditioned medium derived from C2C12 cultures were measured using the Mouse TGF-β1 ELISA DuoSet Kit (R&D Systems) following the manufacturer's instructions. Proliferation and differentiation media not exposed to cells were also measured to determine basal TGF-β1 levels. The results of final secretion from the C2C12 supernatants was calculated by subtracting the basal values of either PM or DM from each sample.

### Cytokine Bead Array (CBA)

Cytokine levels were evaluated by flow cytometry in culture supernatants of infected or uninfected C2C12 cells, in PM or DM at 24 and 120 h of induction. IL-6, IL-10, IL-12p70, TNF, IFN-γ, and MCP-1 were detected using a Cytometric Bead Array (CBA) Mouse Inflammation kit (BD), according to the manufacturer's instructions. Data were acquired using a FACScalibur flow cytometer (BD), and the data analysis was performed by a CBA analysis using the FCAP software (BD).

### Treatments With Conditioned Medium

Conditioned medium (CM) obtained from C2C12 cells, as described in Section TGF-β1 Measurements, was used to treat fresh C2C12 cells. Cells were plated on coverslips in PM. After 24 h of plating, cultures were treated with CM diluted 1:1 in fresh medium (either PM or DM). The medium was replaced daily for 5 days and cells were fixed for immunofluorescence. Untreated controls were maintained either in PM or DM.

### Dual Luciferase Reporter Assay

6 × 10^4^ C2C12 cells/well were cultured on 24-well plates in DMEM containing 10% fetal bovine serum (Gibco) without antibiotics. Twenty-four hours later, cells were transfected with 200 ng TOPFLASH plasmid and 100 ng Tk-Renilla plasmid using FuGENE HD (Promega) at 4:1 ratio. 18 h after transfection, cells were infected with 3.6 × 10^5^ tachyzoite *T. gondii* forms (ME49 strain). After 2 h, cells were washed with simple medium and fresh proliferation medium was added. After 22 h, the medium of half of the cells was switched to DM and/or were treated with 2 μM BIO (CAS Number 667463-62-9, Sigma) for 20 h in order to activate the Wnt/β-catenin signaling pathway. Cells were then lysed using Passive Lysis Buffer (Promega) and Firefly and Renilla luciferase activities were detected according to the manufacturer's protocol (Dual Luciferase Reporter Assay System, Promega).

### Morphometric and Statistical Analyses

At least six microscopic fields were obtained from each experimental condition in three independent experiments, corresponding to a 0.09 mm^2^ area each. The relative differentiation rate was calculated by counting the number of nuclei inside MyHC-positive cells divided by the number of total DAPI positive cells per microscopy field. The relative fusion index was determined as the number of MyHC-positive cells with more than two nuclei and divided by the total number of cells (DAPI-positive) per microscopic field (Joulia et al., 2003). The number of mature myotubes was estimated by the number of MyHC-positive cells that contained at least five myonuclei divided by the number of MyHC-positive cells per field, multiplied by 100. Morphometric analyses of the myotube areas were performed with the Zen Software (Zeiss) using images acquired with a confocal Zeiss microscope (Plataforma de Microscopia Óptica de Luz Gustavo de Oliveira Castro, PLAMOL, UFRJ). The percentage of positive myogenin and MyoD positive nuclei were obtained by dividing the number of positive nuclei by the total number of DAPI positive nuclei per microscopic field and multiplied by 100. Data were analyzed using the GraphPad Prism software version 5.0 for Windows, GraphPad Software, La Jolla California USA, www.graphpad.com. A two-way ANOVA test was used applying Bonferroni's post-test, and changes were considered statistically significant when *p* < 0.05. An unpaired Student's *T-*test was applied to the morphometric analyses, also considering statistically significant changes when *p* < 0.05.

## Results

### *T. gondii* Impairs C2C12 Differentiation and Fusion

**Figure 1:**
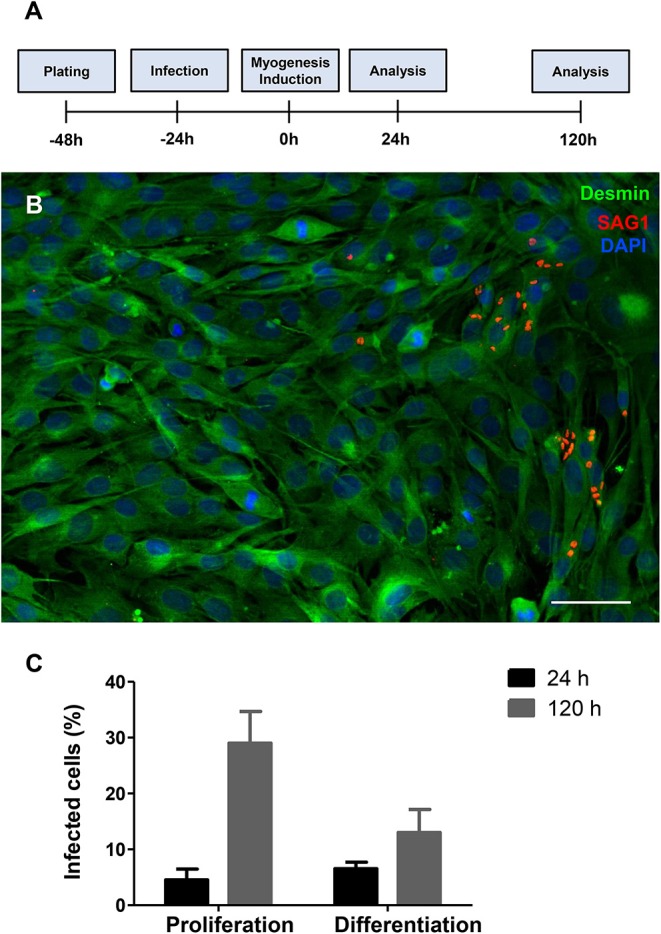
*T. gondii* infection profile in C2C12 cells. The experimental design is shown in **(A)** Cells are plated and infected with tachyzoites after 24 h of plating. Then, half of the cultures have their medium changed to DM. Analyses were carried out 24 and 120 h after myogenesis stimulus with DM. Enrichment in muscle cells was confirmed by desmin staining (**B**, in green) and the presence of tachyzoite forms of *T. gondii* was detected with anti-SAG1 staining (red) as shown in this representative micrograph of infected cells treated with DM for 24 h. Cells were stained with Giemsa stain and the percentage of infected cells was determined by light microscopy. Graphs in **(C)** show the average and standard error of three independent experiments. Scale bar = 50 μm.

C2C12 cells were infected by *T. gondii* as described in the section Method. The establishment of *T. gondii* infection was assessed by light microscopy, in Giemsa-stained cells (Figures S1, S2), and by immunofluorescence to SAG1, a marker for the tachyzoite forms of the parasite (Figure 1). Twenty-four hours post-infection, cells were either maintained in proliferation conditions or switched to differentiation by changing their medium for DM. 24 h later, corresponding to 48 h post infection (hpi), cultures maintained with PM or DM displayed a total of 4.5 ± 3.3 and 6.5 ± 2% of cells bearing parasites, respectively (Figure 1). One hundred and forty-four hours post infection, cultures maintained in PM exhibited 29 ± 9.8% cells containing intracellular parasites, whereas cells in DM displayed 13 ± 7.2% parasitism (*p* < 0.01).

**Figure 2:**
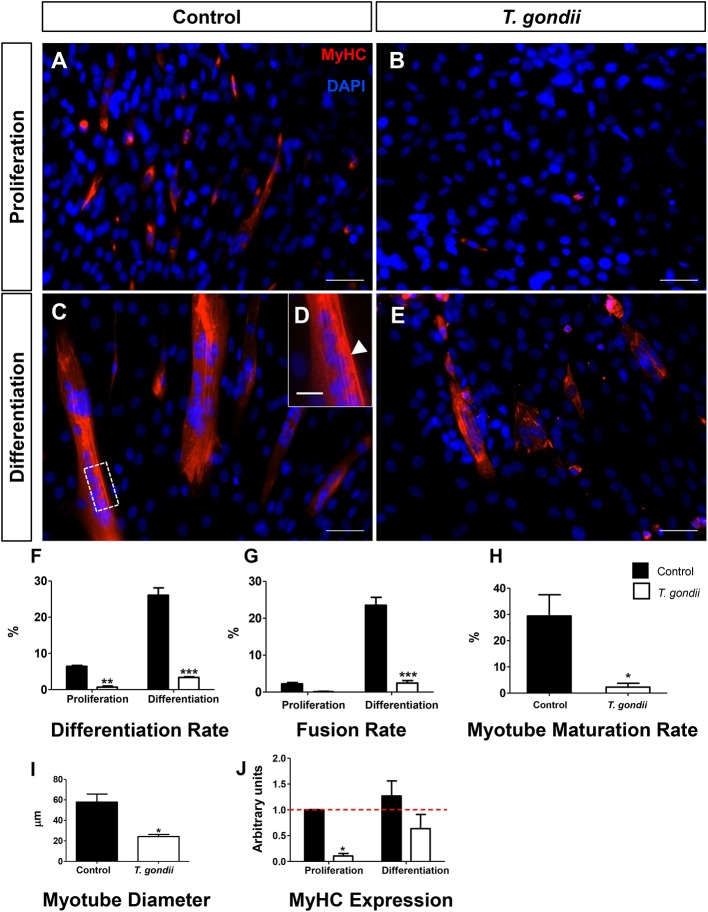
*T. gondii* impairs myogenesis and myotube maturation. C2C12 cells were stained for MyHC, a terminal marker of SkMC differentiation and analyzed by confocal microscopy **(A–E)**. Differentiation was considered in positively MyHC-stained cells **(F)**. Treatment with DM for 5 days greatly increased the number of stained cells from 6 to 26% in uninfected cultures **(F)**. *T. gondii* infection reduced the differentiation rate. Decreases in fusion rates were also observed in *T. gondii*-infected cultures **(G)**, as determined by the number of nuclei within MyHC-positive cells with at least two nuclei. Myotube formation was also impaired by infection in DM treated cultures **(H)**. The deleterious effect of the infection was also reflected in the size of the myotubes **(I)**. Changes in myogenesis induced by the parasite were also observed at the transcriptional level, since MyHC mRNA levels were reduced in infected cultures **(J)**. Results of at least three independent experiments. *p < 0.05, **p < 0.01; ***p < 0.0001, Two-Way ANOVA with Bonferroni post-test. Red dotted line represents the value of control uninfected cultures maintained in Proliferation Medium. Scale bars in **(A–C,E)** = 50 μm, **(D)** = 20 μm.

The impact of *T. gondii* on the capacity of C2C12 cells to differentiate and fuse was evaluated as indicated by Giemsa staining (Figures S1, S2) and MyHC immunostaining (Figure 2), after 120 h. Cells maintained in PM exhibited low levels of differentiation (cells with positive MyHC staining with at least one nucleus), as indicated by <6.4 ± 0.4% MyHC positivity, whereas uninfected dishes maintained in DM reached 26 ± 0.3% of MyHC-stained cells, either mononuclear or multinuclear cells (Figures 2A,C,D). Notably, *T. gondii* infection was highly disruptive to C2C12 differentiation, since infected cultures kept either in PM and DM exhibited only 0.6 and 3.3% MyHC positive stained cells, respectively (Figures 2B,E,F). While uninfected cells in PM exhibited a low basal fusion (2.2%), uninfected DM-treated cultures reached 23%. Infected cultures maintained in DM presented a drastic reduction in the number of fused cells (2.44%, *p* < 0.0001) when compared to uninfected cells maintained in DM (Figure 2G). This reduction in fusion rates led to a proportional decrease in the number of mature myotubes in infected cultures (2.2 vs. 29.4% in uninfected controls, *p* < 0.05, Unpaired Student's t test, Figure 2H). Myotubes found in infected cultures also displayed decreased diameter (57.8 vs. 24.2 μm, *p* < 0.05, Unpaired Student's *t*-test, Figure 2I). To confirm that *T. gondii* infection impairs myocyte differentiation, RT-qPCR for *myosin heavy chain* was performed. Five days after myogenesis induction, DM-treated cultures exhibited a slight, yet not statistically significant, increase in *MyHC* expression when compared to PM (1.3-fold, *p* > 0.05). *T. gondii*-infected dishes showed a drastic down-regulation of *MyHC* expression, both in PM (90%, *p* < 0.05) and DM (63%, *p* > 0.05) (Figure 2J).

### Infection Alters MRFs Expression/Immunoreactivity

The influence of *T. gondii* infection on the expression and immunolocalization of myogenic regulatory transcription factors (MRFs) MyoD, myogenin, Myf5, and Mrf4 (Myf6) was assessed on C2C12 cells.

**Figure 3:**
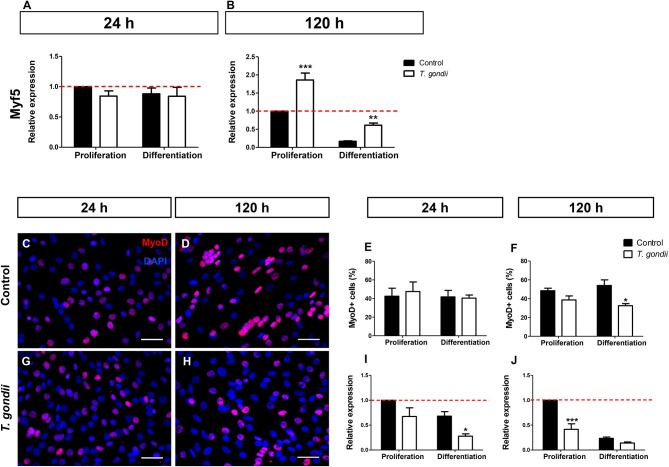
Infection affects early myogenic gene expression. Relative expression of Myf5 was analyzed by RT-qPCR. Myf5 levels were determined at the initial time of myogenesis (24 h, **A**) and at the latest time point (120 h, **B**). Uninfected and infected cells in PM or DM were immunostained for MyoD (red) and DAPI (blue) at 24 **(C,G)** and 120 h of induction **(D,H)**. Quantification of MyoD-positive cells at 24 h indicate that infection led to no significant changes in the number of MyoD cells **(E)**. After 120 h of myogenic stimulation, infected-DM cultures displayed 32% of MyoD-positive cells, whereas in uninfected DM dishes a 54% rate was observed **(F)**. RT-qPCR revealed that at 24 h of induction, MyoD transcript was significantly less abundant in infected-DM dishes, when compared to the controls **(I)**. During late stage myogenesis (120 h), *T. gondii*-infected cells in PM presented a 59% decrease in MyoD expression when compared to controls **(J)**. Results of at least three independent experiments *p < 0.05, **p < 0.01; ***p < 0.0001, Two-Way ANOVA with Bonferroni post-test. The red dotted line represents the value of control uninfected cultures maintained in the Proliferation Medium. Scale bar = 50 μm.

Myf5, expressed in committed satellite cells and myoblasts showed no change after 24 h of culture in DM (Figure 3A). However, after 120 h, non-infected cultures maintained in DM exhibited an 83% decrease in Myf5 expression, as indicated by RT-qPCR (Figure 3B). Interestingly, at this time point *T. gondii*-infected cultures displayed higher Myf5 levels when compared to their respective controls (1.85-fold in PM and 3.58-fold in DM, *p* < 0.0001 and *p* < 0.01, respectively, Two-Way ANOVA with Bonferroni post-test), confirming their immaturity regarding myogenesis.

Next, the presence of MyoD, an activated myoblast and myocyte marker, was investigated. No changes in MyoD immunostaining were detected in infected dishes after 24 h of differentiation (48 hpi) when compared to non-infected cultures (Figures 3C,G,E). However, at 120 h, the number of MyoD-positive cells were decreased by 21% (*p* > 0.05) and 40% (*p* < 0.05, Two-way ANOVA, with Bonferroni post-test) in infected cultures maintained in PM and DM, respectively, when compared to uninfected ones (Figures 3D,H,F). The RT-qPCR analysis confirmed altered MyoD expression after *T. gondii* infection. After 24 h of differentiation in DM, uninfected cultures showed no significant alteration in MyoD expression when compared to PM, although a decreasing trend was observed (Figure 3I). At this time point, *T. gondii* infection induced a decrease in MyoD expression in cultures maintained in PM (33%, *p* > 0.05) and in DM (60%, *p* < 0.05, Two-way ANOVA, with Bonferroni post-test) when compared to uninfected cultures at 24 h (Figure 3I). The same effect was observed at 120 h of myogenesis induction. *T. gondii*-infected cultures displayed a 59% (*p* < 0.0001) and 40% (*p* > 0.05) decrease when compared to their respective uninfected cultures in PM and DM, respectively (Figure 3J).

**Figure 4:**
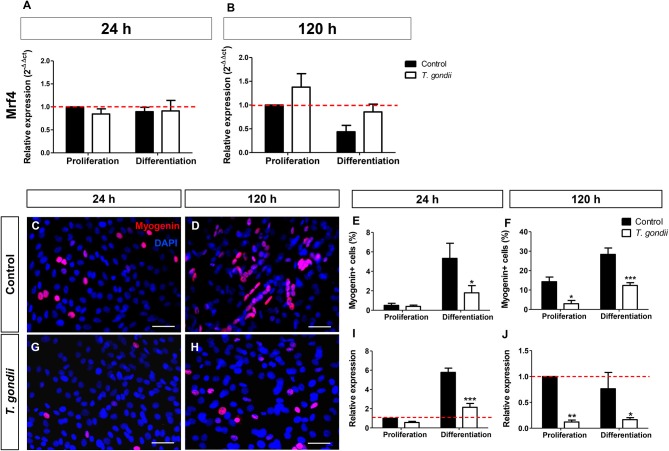
Analysis of late myogenic genes expression in *T. gondii*-infected C2C12 cultures. After 24 h **(A)** and at 120 h **(B)** of myogenic stimulation Mrf4 expression was assessed by RT-qPCR and no significant changes were observed. Uninfected and infected cells in PM or DM were immunostained for myogenin (red) at 24 **(C,G)** and 120 h in DM **(D,H)**. The cell nucleus was stained with DAPI (blue). At 24 h of induction, uninfected cultures treated with DM showed significant increase in the number of myogenin-positive cells **(E)**, accompanied by a 6-fold increase in myogenin gene expression **(I)**. Infected cultures failed to show myogenin immunoreactivity at 24 h **(G,E)**, as well as mRNA expression **(I)**. 120 h after the initiation of the myogenic stimulus, *T. gondii* significantly reduced number of myogenin-positive cells **(F)** and expression **(J)**. Results of at least three independent experiments. *p < 0.05, **p < 0.01; ***p < 0.0001, Two-Way ANOVA with Bonferroni post-test. Red dotted line represents the value of control uninfected cultures maintained in Proliferation Medium. Scale bar = 50 μm.

*Mrf4*, expressed only in later stages of the myogenic process, was analyzed by RT-qPCR. The levels of *Mrf4* transcripts in our cultures were low, with CT values near 35. No significant changes in *Mrf4* relative expression were verified in our cultures (Figures 4A,B).

Finally, the expression and immunoreactivity of *myogenin* in C2C12 cells was evaluated. At 24 h of differentiation in DM, 5% of the uninfected cells were *myogenin*+ while *T. gondii*-infected cultures showed only 2% of positivity (*p* < 0.05, Two-Way ANOVA, with Bonferroni post-test, Figures 4C,G,E). At 120 h of differentiation, this number increased to 28.35% when compared to cells in PM (14.3%) (Figures 4D,H,F). *T. gondii* infection induced a strong inhibition of *myogenin* immunoreactivity at 120 h of differentiation (144 hpi). Infected cultures in PM displayed 2.96% of *myogenin*-positive nuclei and those kept in DM showed only 12.34% positivity. This observation was confirmed by RT-qPCR, indicating that uninfected cells in DM exhibited a 5.7-fold increase in *myogenin* expression at 24 h induction (Figure 4I), while infected C2C12 cultures presented decreased *myogenin* expression when compared to their correspondent controls (44%, *p* > 0.05 in PM and 63%, *p* < 0.0001 in DM, Two-Way ANOVA, with Bonferroni post-test, Figure 4I). At 120 h, *T. gondii* infection greatly reduced the level of myogenin transcript in both conditions (88% in PM, *p* < 0.01 and 78% in DM, *p* < 0.05, Two-Way ANOVA with Bonferroni post-test, Figure 4J).

### *T. gondii* Infection Leads to a Proliferative, Undifferentiated State of C2C12 Cells

**Figure 5:**
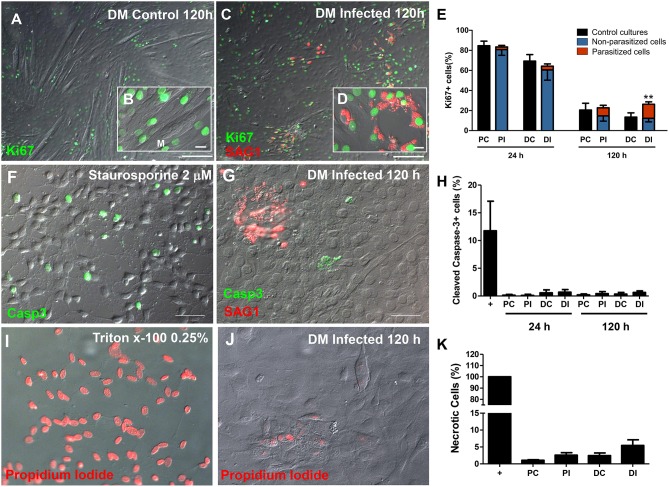
The C2C12 proliferation rate was altered by infection. Ki67 immunostaining was used as parameter to determine the number of mitotic cells. Micrographs depict Ki67 staining of cultures in DM after 120 h of induction in uninfected **(A,B)** and *T. gondii*-infected dishes **(C,D)**. 24 h after treatment with DM no changes were detected **(E)**. *T. gondii* increased the proliferation of DM-treated cultures, when compared to uninfected dishes **(E)**. No Ki67 staining was observed in myotubes (M, in **B**), whereas cells harboring tachyzoites (shown in more detail in **D**) displayed intense nuclear immunolabeling. Cell death was assessed by staining for cleaved caspase-3 for apoptosis **(F–H)** and by propidium iodide uptake experiments for necrosis **(I–K)**. *T. gondii* infection did not induce apoptotic cell death, since caspase-3 stained cells ranged from 0.1 to 0.7% in all experimental conditions **(H)**. Treatment with Staurosporine 2 μM in DMEM high glucose with no serum for 2 h was used as a positive control **(F)**. Tachyzoites were detected with anti-SAG1 antibody and displayed no correlation to the presence of caspase-3-stained cells **(G,H)**. Necrosis was calculated by the percentage of PI-stained nuclei. Triton x-100 0.25% was used as positive control **(I)** and led to 100% of stained cells **(K)**. Infected C2C12 cells at 120 h of myogenesis induction **(J)** displayed no significant difference when compared to uninfected cultures, both in PM or DM. *N* = 4, **p < 0.01, Two-Way ANOVA with Bonferroni post-test. Scale bars: 100 μm in **(B,D)**, 20 μm in **(C,E)**.

Following the observations that infected cultures exhibited altered MRF expression patterns and, consequently, decreased myotube formation, we investigated whether the infection also altered C2C12 cell proliferation using the proliferation marker Ki67. Cells maintained in PM for 24 h exhibited an average of 84.6 ± 9% Ki67-positive cells. At 24 h of induction with DM a slight, yet non-significant, decrease in the proportion of Ki67-positive cells (69.3 ± 12%) was detected (Figure 5), and infected cultures displayed comparable proliferation rates (82.4 ± 11 in PM and 63 ± 20% in DM). At 120 h, non-infected cultures in both PM and DM presented less Ki67 staining than non-infected cultures at 24 h, reaching 26 ± 9 and 11.7 ± 3% of the total cellular population, respectively. As expected, fully differentiated myotubes did not show positive staining for Ki67 (Figure 5A). Infected dishes kept in PM for 120 h exhibited 28.5 ± 9% of proliferative cells (Figure 5E), very similar to what was observed in the non-infected controls at this same time point. However, Ki67 positivity reached 29.8 ± 14% (*p* < 0.01, Two-Way ANOVA, with Bonferroni post-test) in infected cultures kept in DM when compared to uninfected dishes in DM, indicating a ~2.5-fold increase in the number of proliferative cells (Figure 5E). A differential quantification of Ki67-positive staining in infected dishes was performed in order to determine whether cells harboring parasites would be preferentially proliferating, or if a bystander effect would be involved in increased proliferation. In infected C2C12 cultures maintained in DM for 120 h, 46% of Ki67-stained cells corresponded to parasitized cells (14.4% out of 29.8%, Figure 5E, red bars).

In order to exclude the possibility that increased proliferation could be due to a compensatory mechanism in response to parasite-induced cell death, the cultures were stained for cleaved caspase-3, a classic effector apoptosis marker (Nicholson et al., 1995). Staurosporine at 2 μM was used as a positive control for 2 h in uninfected cultures and presented an average of 11.75% of caspase-3 staining. The different C2C12 treatments (differentiation and infection) did not lead to changes in apoptosis levels (Figures 5F,H). Host cell necrosis was assessed by permeability to propidium iodide, which indicates loss of membrane integrity. Triton x-100 0.25% was used as the positive control for 5 min and led to positive staining in 100% of cells (Figures 5I,K). Uninfected cultures in PM presented 1.07% cells with positive PI staining, whereas this number reached 2.44% in uninfected DM-treated cultures (*p* > 0.05, One Way ANOVA with Bonferroni post-test). Infected cultures displayed a slight, albeit non-statistically significant, increase in the number of PI positive cells (Figures 5I,K).

### *T. gondii*-Infected C2C12 Cells Display an Altered Secretory Pattern

*T. gondii* infection is known to modulate host cell responses and induce an inflammatory milieu that can generate paracrine effects in the cell culture. CBA was used to determine which cytokines and chemokines were released during the infection and which may, therefore, influence the myogenic process. Among the tested factors (IL-6, IL-10, IL-12p70, IFN-γ, TNF, and MCP-1), only IL-6 and MCP-1 were detected as secreted.

**Figure 6:**
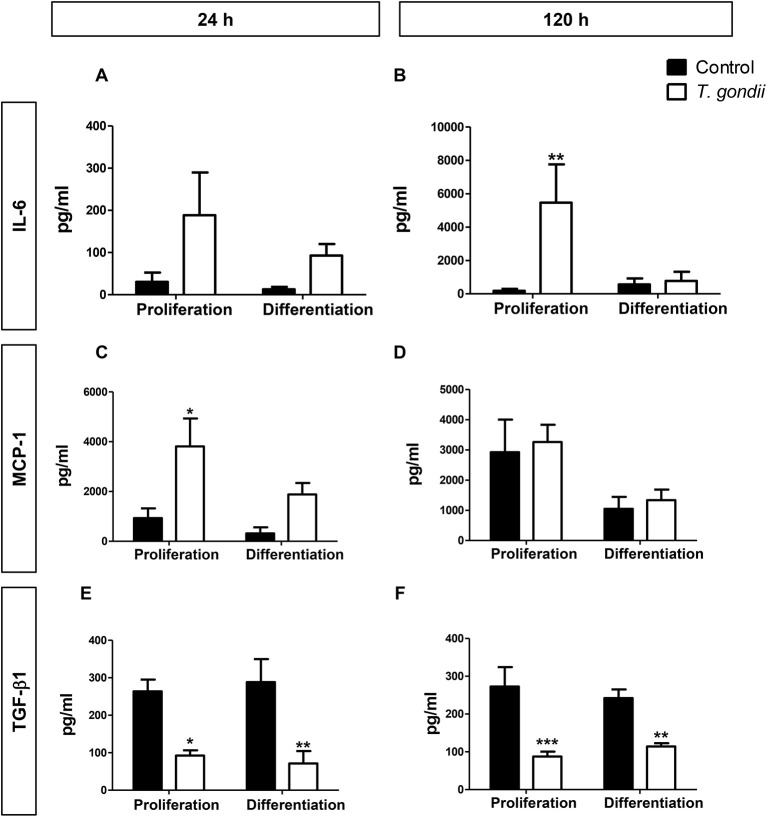
Secretory profile during *T. gondii* infection. Conditioned medium from C2C12 cells was assayed for INF-γ, TNF-α, IL-10, IL-12p70, IL-6, and MCP-1 with CBA assay. The experimental design in shown in **(A)**. IL-6 **(B)** and MCP-1 **(C)** were greatly increased with *T. gondii* infection, whereas a decrease of TGF- β1 secretion was observed at both evaluated times, as assesses by ELISA **(E,F)**. MCP-1 remained unaltered at 120 h of myogenesis **(D)**. Results of at least three independent experiments. *p < 0.05, **p < 0.01; ***p < 0.0001, Two-Way ANOVA with Bonferroni post-test.

At 48 hpi, infected cells maintained in PM exhibited a 20-fold increase in IL-6 (*p* > 0.05, Figure 6A) and a 4-fold increase in MCP-1 (*p* < 0.05, Two-Way ANOVA, with Bonferroni post-test) secretion when compared to uninfected cultures in PM (Figure 6C). Infected C2C12 cells maintained in DM for 24 h also displayed increased levels of secreted IL-6 compared to non-infected cells (7-fold, *p* > 0.05, Figure 6A) and MCP-1 levels were increased by 6.8-fold (*p* > 0.05) (Figure 6C). IL-6 was greatly increased in infected cells in PM at 144 hpi (28,89-fold, *p* < 0.01, Two-Way ANOVA, with Bonferroni post-test) but not in DM (Figure 6B). MCP-1 levels in infected cultures at 120 h remained comparable to uninfected cultures (Figure 6D).

TGF-β1 is an anti-inflammatory cytokine known to greatly inhibit myogenesis in C2C12 cells (Massagué et al., 1986; Olson et al., 1986). We hypothesized that TGF-β1 secretion could be the mechanism through which *T. gondii* impaired myogenesis. However, we observed that this cytokine was greatly reduced in the supernatant of infected cultures, at all assessed times (Figures 6E,F). Regardless of the culture medium, infected dishes presented TGF-β1 secretion ranging from 71 to 114 pg/ml, while TGF-β1 concentrations ranged between 242 and 288 pg/ml in uninfected cultures.

**Figure 7:**
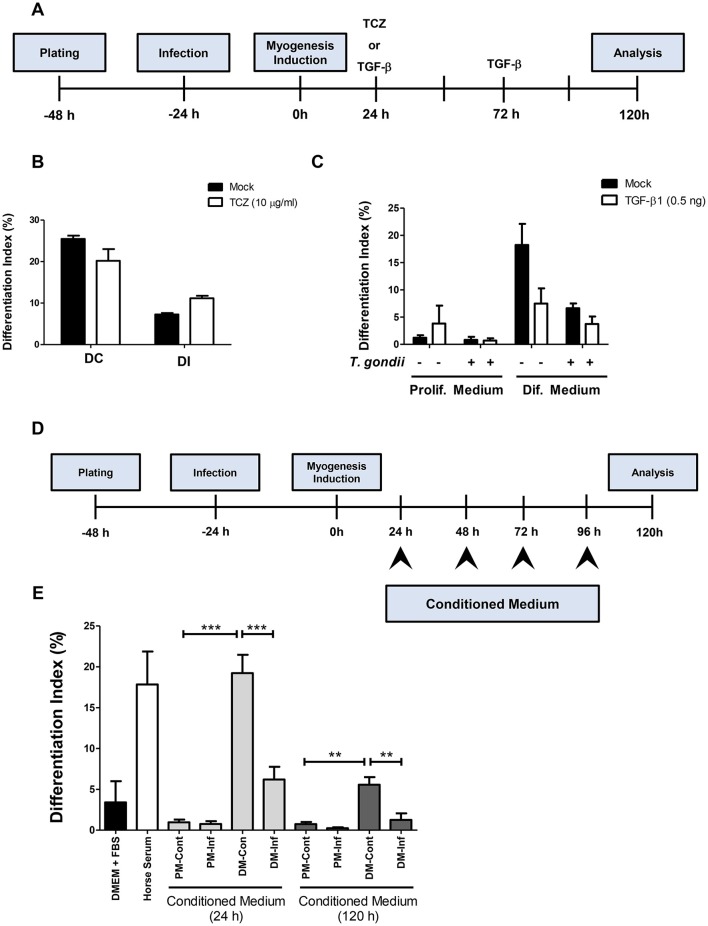
Soluble factors released from infected cultures have an impact on myogenesis. In order to test whether increased IL-6 or decreased TGF-β1 played a role on impairment of myogenesis, C2C12 were infected and treated with Tocilizumabe **(B)** or recombinant TGF-β1 **(C)**. The experimental designs are shown in **(A,D)**. TCZ had no significant impact on differentiation rate in cultures maintained in DM **(B)**. TGF-β decreased the differentiation rate, as shown by the number of MyHC-positive cells per field in both PM and DM **(C)**. Conditioned medium (CM) from infected C2C12 cells was used to treat fresh myoblasts. CM from DM-treated cultures (DM-Cont) 24 and 120 h increased the differentiation rate **(E)**, whereas DM-Inf 24 and 120 h had an opposite effect, reducing myogenesis. Results of at least three independent experiments. **p < 0.01; ***p < 0.0001, One-Way ANOVA with Bonferroni post-test.

In order to determine whether increased IL-6 or decreased TGF-β secretion plays a role in myogenesis impairment in C2C12 cells, treatments with 10 μg/ml Tocilizumabe (TCZ), a neutralizing antibody that inhibits the IL-6 receptor and with recombinant TGF-β1 (rTGF, 0.5 ng) were performed (Figure 7A). TCZ had no impact on myogenesis rates and myotube formation in uninfected cultures (Figure 7B). Treatment with TCZ of *T. gondii*-infected cultures led to no significant alteration in the number of MyHC-positive cells and myotubes (Figure 7B). rTGF addition caused no alteration in the number of MyHC-positive cells in PM-treated cultures (Figure 7C), although a negative effect on myogenesis in both uninfected and infected DM-treated cultures was observed, with reduced numbers of MyHC-positive cells (Figure 7C).

Since neither IL-6 nor TGF-β seem to be directly involved in defective myogenesis in infected cultures, conditioned medium transfer experiments were carried out. Uninfected C2C12 cells were treated for 5 days with a 1:1 mixture of conditioned medium with fresh medium (either PM or DM, Figure 7D). Cells treated with CM from uninfected or infected cultures maintained in PM for 24 h (PM-Cont and PM-Inf) presented 0.96 and 0.76% of MyHC-positive cells, respectively (Figure 7E). Cultures treated with DM-Cont 24h displayed differentiation rates similar to that observed in cultures maintained with DM alone (19.2%), whereas treatment with CM from DM-Inf 24h indicated 6.2% MyHC-positive cells (*p* < 0.0001, One-Way ANOVA with Bonferroni post-test). The same effect was observed in cultures treated with CM from DM-Cont 120 h, which displayed 5.6% of MyHC cells, vs. 1.25% found in DM-Inf 120h-treated dishes (*p* < 0.01, One-Way ANOVA, with Bonferroni post-test, Figure 7E).

### Wnt/β-Catenin Pathway Activation Is Impaired by *T. gondii*

**Figure 8:**
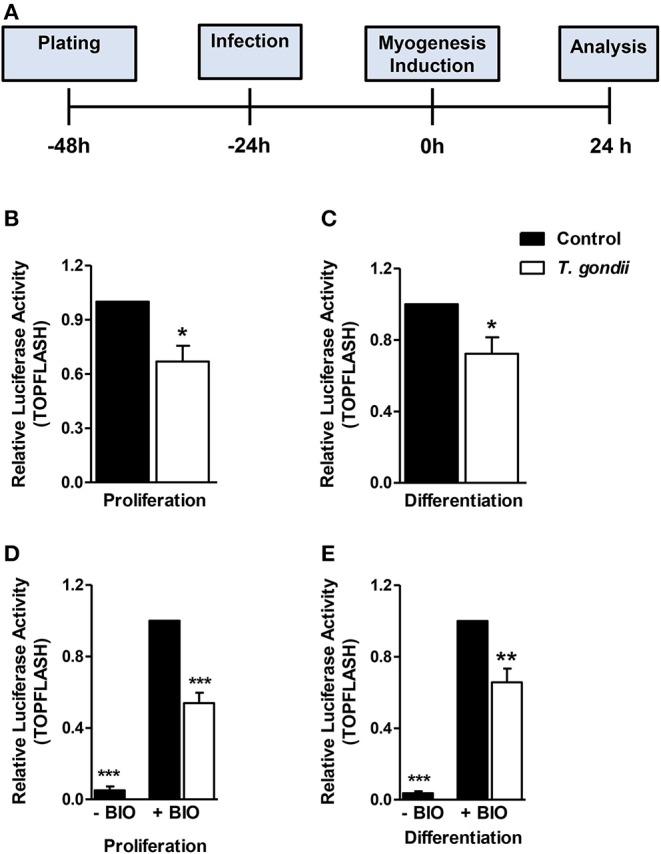
Wnt/β-catenin pathway is decreased in *T. gondii*-infected cultures. The experimental design in shown in **(A)**. Luciferase assay for TCF/LEF promoter activation by β-catenin showed that *T. gondii*-infected cultures at 24 h of induction displayed significantly lower luciferase signal when compared to controls **(B,C)**. BIO treatment significantly increased luciferase activity in uninfected controls **(D,E)**. However, *T. gondii* infection impaired such effect. *p < 0.05, **p < 0.01, ***p < 0.001, unpaired Student's T test. Scale bars: 20 μm.

Since *T. gondii* infection altered MRFs expression and cytokine secretion at times as early as 24 h of induction (corresponding to 48 hpi), we investigated an upstream myogenesis regulating pathway, the Wnt/β-catenin pathway (Figure 8A). The effect of the infection on the activation of the Wnt/β-catenin pathway was confirmed by dual luciferase reporter assays for the TCF/LEF reporter. Infected cultures maintained in PM presented a 33% reduction in luciferase activity when compared to controls (Figure 8B, *p* < 0.05, unpaired Student's *T*-test). In addition, a significant decrease was observed in infected DM-treated cultures, when compared to uninfected DM-treated controls (Figure 8C, *p* < 0.05, unpaired Student's *T*-test). BIO, a selective pharmacological GSK3 inhibitor and, therefore a Wnt/β-catenin pathway activator, was used to confirm these findings. Indeed, luciferase activity increased ~25-fold in uninfected cultures treated with PM and DM (Figures 8D,E, *p* < 0.001 unpaired Student's T test). This effect was impaired in *T. gondii*-infected cultures by 46 and 34% in PM and DM-treated cultures, respectively (Figures 8D,E, *p* < 0.0001 and <0.01, respectively). However, the overall content of β-catenin remained unaltered in *T. gondii*-infected cultures (data not shown).

## Discussion

*T. gondii* displays an interesting interaction with the skeletal muscle system, in which tissue cysts are formed (Dubey, 1998). Such tropism is important for the transmission cycle of the parasite, since predation of infected prey by felids may favor the sexual cycle (Dubey and Frenkel, 1972). However, the acquired infection can cause damages to the skeletal muscle in intermediate hosts, and clinical reports have demonstrated that *T. gondii* infection may cause intense myositis, electromyographic abnormalities and muscle pain (Montoya et al., 1997; Hassene et al., 2008; Cuomo et al., 2013). We used the mouse myoblast cell line C2C12 to investigate the mechanism by which *T. gondii* infection may impact skeletal muscle physiology. Previous data from our laboratory using primary skeletal muscle cell cultures have demonstrated that infection with the highly virulent RH strain of the parasite reduced the number of multinucleated cells (Gomes et al., 2011). We chose the type II strain ME49 that exhibits reduced virulence compared to the laboratory-adapted RH strain, thus avoiding the confounding factor of high levels of host cell lysis by the latter (Kirkman et al., 2001). Moreover, previous observations from our group showed that vertical transmission of *T. gondii* induces alterations in the fetal myogenesis (Gomes and Barbosa, 2016). Low levels of parasitism were detected when C2C12 cells were infected with ME49 tachyzoites (5% at 48 hpi and 10-30% at 144 hpi). However, the number of MyHC+ cells and myotubes were drastically reduced, thus confirming that this infection displays a more general deleterious effect on the differentiation of the C2C12 cell population, despite the low infectivity rate. This deleterious effect on myogenesis was also observed in proliferating cells, which display a basal spontaneous myogenic induction, due to high cellular density (Tanaka K. et al., 2011).

Myogenesis, characterized by myocyte differentiation and fusion (Dedieu et al., 2002; Berendse et al., 2003) is essential in muscle development, after birth for breathing and for muscle growth, and also in adults for muscle regeneration, following injury (Le Grand and Rudnicki, 2007). In order to gain insights into the molecular mechanisms through which *T. gondii* impairs myotube formation, we investigated the expression levels of the main MRFs: Myf5, MyoD, Mrf4 and myogenin. Myf5 is a transcription factor that, along with MyoD, is activated and expressed in early myogenic program steps (Rudnicki et al., 1993). We found Myf5 transcripts to be decreased in uninfected cultures maintained in DM for 120 h when compared to cells kept in PM, in accordance with known Myf5 decreased expression after commitment to differentiation (Zammit et al., 2006). *T. gondii*-infected cultures presented higher levels of Myf5 when compared to their respective controls, suggesting a delay in the myogenic program of these cells.

It is known that MyoD expression is capable of initiating the myogenic program, even in non-muscle cells (Davis et al., 1987; Weintraub et al., 1989). MyoD targets are related to differentiation, such as myogenin, but also to the cell cycle, such as Ankrd2, Cdkn1c, and calcyclin (Bean et al., 2005), which suggested that proliferation or differentiation pathways are mutually exclusive during myogenesis, and one depends on inhibition of the other. We demonstrated that higher amounts of proliferating cells are found in infected cultures, but it is unclear if the cell cycle itself is affected by *T. gondii* infection. MyoD reduction at the protein level could affect cell proliferation by decreasing myogenin expression, one of its known targets (Buckingham and Rigby, 2014). However, increased Myf5 expression together with decreased MyoD expression suggests that myoblasts in infected cultures are kept in a proliferating myogenic precursor state.

TGF is part of a family of pluripotent growth factors involved in diverse physiological processes, including myogenesis (Liu et al., 2001). During the maturation of C2C12 myotubes, bone morphogenetic proteins (BMPs) are gradually down-regulated, whereas TGF-β (1, 2, and 3) are up-regulated (Furutani et al., 2011). TGF-β1 presents a deleterious effect on myogenesis (Olson et al., 1986), and it has been demonstrated that *T. gondii* infection induce TGF-β secretion in macrophages (Bermudez et al., 1993). However, our data indicate that infected C2C12 cells display reduced TGF-β1 secretion. This behavior was also observed by our group after *T. gondii* infection of neural progenitors (Adesse et al., 2018). Regarding muscle cells, Swierzy et al. (2014) previously demonstrated *T. gondii* infection effects on the TGF-β mRNA expression of myoblasts and myotubes. In that study *T. gondii* infection with the NTE strain (also type II) did not alter TGF-β gene expression. Infected cultures were treated with rTGF, which did not rescue the myogenesis defect. This finding indicates that TGF-β1 found in the supernatant of uninfected cultures may be a marker of differentiated myocytes/myotubes and its decreased secretion in infected cultures may be only the indication that cells remained undifferentiated.

IL-6 is a myokine (Pedersen et al., 2003) and its secretion is increased in muscle cells following exercise acting in physiological processes, not only in skeletal muscle but also systemically (Forcina et al., 2018). However, excessive IL-6 levels can lead to an acute inflammatory response. In this scenario, muscular atrophy and satellite cell exhaustion may occur, leading to tissue inflammation and increased ROS production, along with insulin resistance and possible chronic inflammation (Visser et al., 2002; Haddad et al., 2005; Carson and Baltgalvis, 2010). Pelosi et al. (2014) demonstrated that treatment with IL-6 impaired C2C12 myogenesis, with myogenin and MyHC downregulation, whereas MyoD and Pax7 levels remained unaltered. Treatment with Tocilizumabe, a neutralizing antibody for IL-6R, had no effect on myogenesis rescue in infected cultures. This suggests that IL-6 may not be the major soluble component involved in myogenesis defects induced by *T. gondii*. Another explanation would be that IL-6-mediated defect could act via other receptors that are not blocked by TCZ. Other cytokines and chemokines known to be up-regulated by *T. gondii* infection not included in the CBA kit may also play an important role in controlling the myogenic process in infected cultures. This idea was reinforced by the fact that conditioned medium from infected C2C12 cultures also impaired myogenesis. Indeed, extracellular vesicles released by *T. gondii* or by *T. gondii*-infected cells led to increased proliferation of rat myoblasts (Kim et al., 2016; Li et al., 2018).

*T. gondii* possesses effector proteins that act on host cells, which are capable of inducing the activation of the STAT3, STAT6, and NF-κB pathways (Hakimi et al., 2017), thus leading to the production of many cytokines and immunomodulatory molecules that may interfere in infection latency. It has been demonstrated that the *T. gondii* type II strain could induce the NF-κB pathway through release of the GRA15 protein (Rosowski et al., 2011). As IL-6 and MCP-1 are known NF-κB targets (Libermann and Baltimore, 1990; Shoelson et al., 2006) this effect could be the trigger that leads to the myogenic defects observed in the present model. Since a pro-inflammatory profile can favor myoblast proliferation (Arnold et al., 2007), the environment induced by *T. gondii* infection may promote proliferation, leaving myoblasts unresponsive to myogenic stimulus. Moreover, *T. gondii* effector proteins were also shown to affect c-Myc (Franco et al., 2014) and p21 (Chang et al., 2015) expression, two proteins that regulate host cell proliferation and might explain the increase in Ki67-positive cells found in our system.

The Wnt/β-catenin pathway is one of the regulators of the myogenic program acting on the switch from proliferation to differentiation in SkMCs (Tanaka S. et al., 2011; von Maltzahn et al., 2012). The results presented herein indicate that infected cultures presented reduced β-catenin activation despite the maintenance of global β-catenin contents, as shown by luciferase assays, thus indicating that infection impairs endogenous β-catenin activation, followed by its correct translocation to the myonucleus. β-catenin directly binds cadherins, linking this junctional complex to the actin cytoskeleton. Interestingly, M-cadherin transcripts and protein levels have been shown to be down-regulated on *T. gondii* infection of muscle cells as early as 3 and 24 h post infection, respectively (Gomes et al., 2011). Indeed, M-cadherin down-regulation is capable of reducing myogenesis through reduction in active β-catenin, thus resulting in decreased myogenesis (Wang et al., 2013). Since myogenesis induction began at 24 hpi, it is suggested that the cadherin-catenin complex is already dismantled and, therefore, cells cannot respond to Wnt activation. The observation that *T. gondii* infection inhibits BIO-induced activation of β-catenin pathway indicates that this effect occurs downstream of the β-catenin destruction complex (MacDonald et al., 2009).

**Figure 9:**
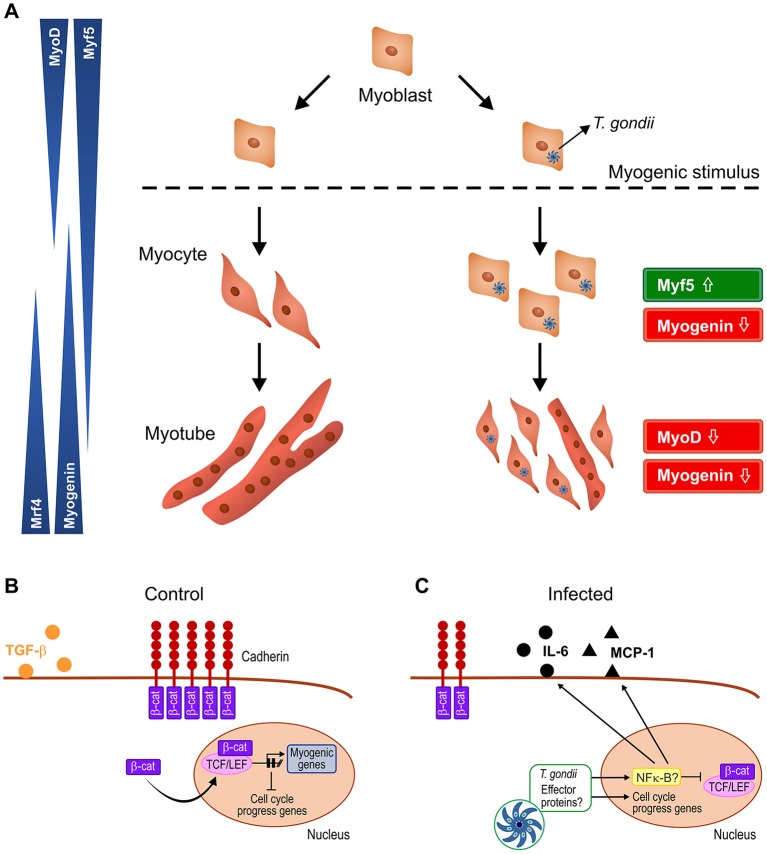
Schematic representation of the proposed mechanism by which *T. gondii* affects myogenesis. Upon serum withdrawal myoblasts begin the myogenic process, differentiating into myocytes and expressing MRFs in a coordinated fashion, leading to formation of mature myotubes, whereas infected myoblast cultures display undifferentiatiated cells and smaller myotubes **(A)**. The Wnt/β-catenin pathway is activated in uninfected cultures, shutting down cell proliferation and inducing myogenesis, with TGF-β production **(B)**. *T. gondii* infection leads to cadherin down-regulation, thus destabilizing the cadherin-catenin complex, inducing β-catenin destruction **(C)**. In addition, intracellular parasites release effector proteins that translocate to host cell nucleus and direct the expression of inflammation-related genes such as NF-κB, increasing secretion of pro-inflammatory molecules (IL-6 and MCP-1) and reducing anti-inflammatory cytokine TGF-β1. Hence, infected cultures remain highly proliferative, regardless of parasitism rates.

In summary, our results point to a disruptive effect of *T. gondii* on C2C12 myogenesis, creating a pro-inflammatory milieu that spreads to neighboring cells and impairs their response to myogenic stimuli (Figure 9). These findings are relevant in the context of congenital and acquired infection and may shed light on the impact of this parasite on muscle physiology.

## Data Availability Statement

The datasets generated for this study are available on request to the corresponding author.

## Author Contributions

DA and HB conceptualization, supervision, and funding acquisition. DA and PV methodology, data curation, and writing—original draft. DA, HB, VM, and PV validation. DA, PV, MW, DB, and DP formal analysis. PV, MW, DB, DP, and JA investigation. DA, HB, MW, VM, GB-B, and JA resources. HB, MW, and VM writing—review & editing. DA visualization and project administration.

### Conflict of Interest

The authors declare that the research was conducted in the absence of any commercial or financial relationships that could be construed as a potential conflict of interest.

## Supplementary Material

Figure S1 Culture evaluation with Giemsa stain after 24 h of myogenesis induction. **(A,B)** PM-treated cells, **(C,D)** DM-treated cells. Infected cultures are on the right panels. Parasites are indicated by white arrows. Scale bars: 200 μm. Insets in **(C,D)** 20 μm.

Figure S2 Culture evaluation with Giemsa stain after 120 h of induction of myogenesis. **(A,B)** PM-treated cells, **(C,D)** DM-treated cells. Infected cultures are on the right panels. Parasites within parasitophorous vacuoles are indicated by arrows and cyst-like structures are indicated by asterisks (^*****^). Scale bars: 200 μm. Insets: 20 μm.
