# Supplementary figures and images for: Toxoplasma gondii Impairs Myogenesis in vitro, With Changes in Myogenic Regulatory Factors, Altered Host Cell Proliferation and Secretory Profile
Source: Front Cell Infect Microbiol. 2019 Nov 27;9:395. doi: 10.3389/fcimb.2019.00395 (PMC6890860; doi:10.3389/fcimb.2019.00395)

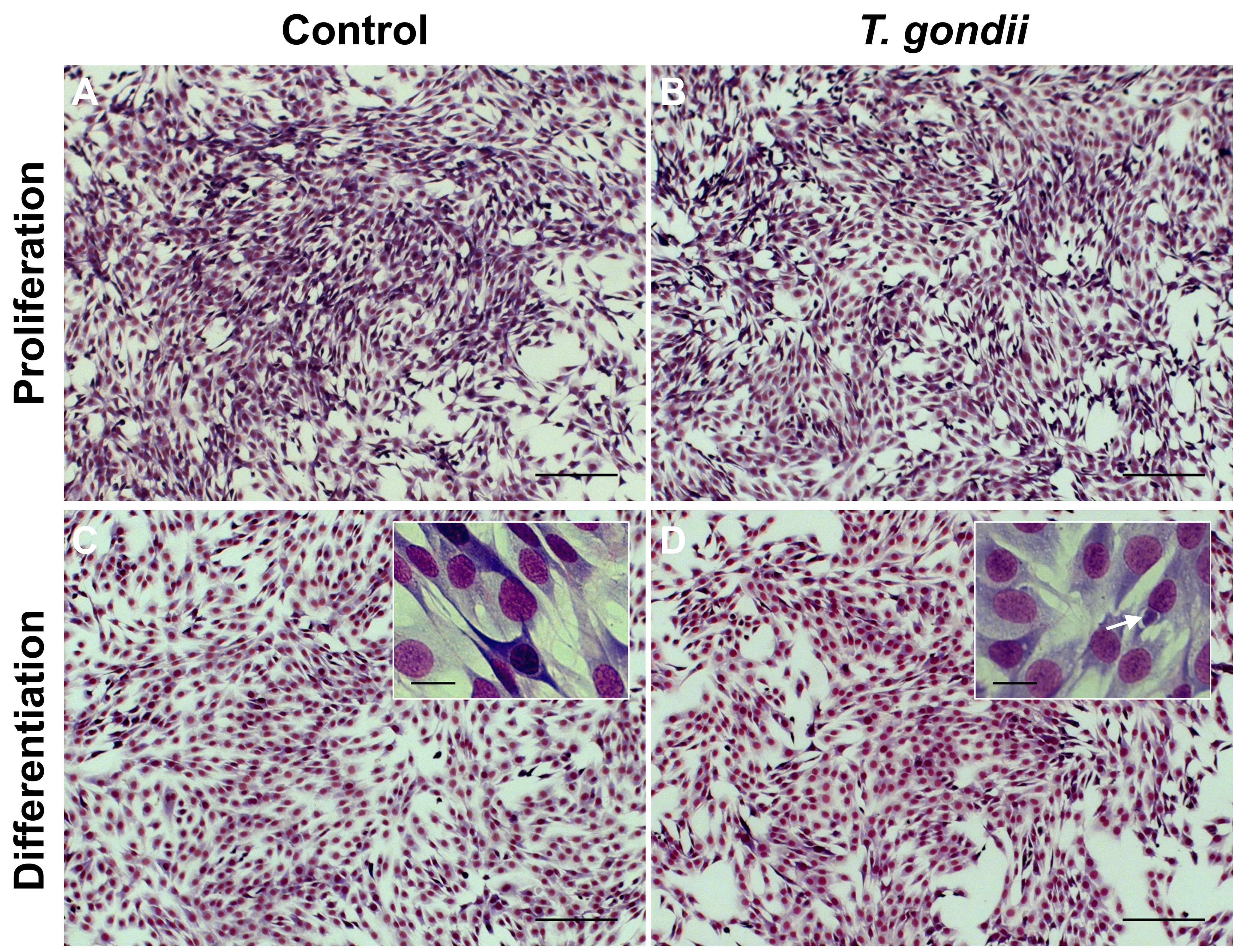

Supplement: Figure S1 — Culture evaluation with Giemsa stain after 24 h of myogenesis induction. (A,B) PM-treated cells, (C,D) DM-treated cells. Infected cultures are on the right panels. Parasites are indicated by white arrows. Scale bars: 200 μm. Insets in (C,D) 20 μm. [file Image_1.TIF]

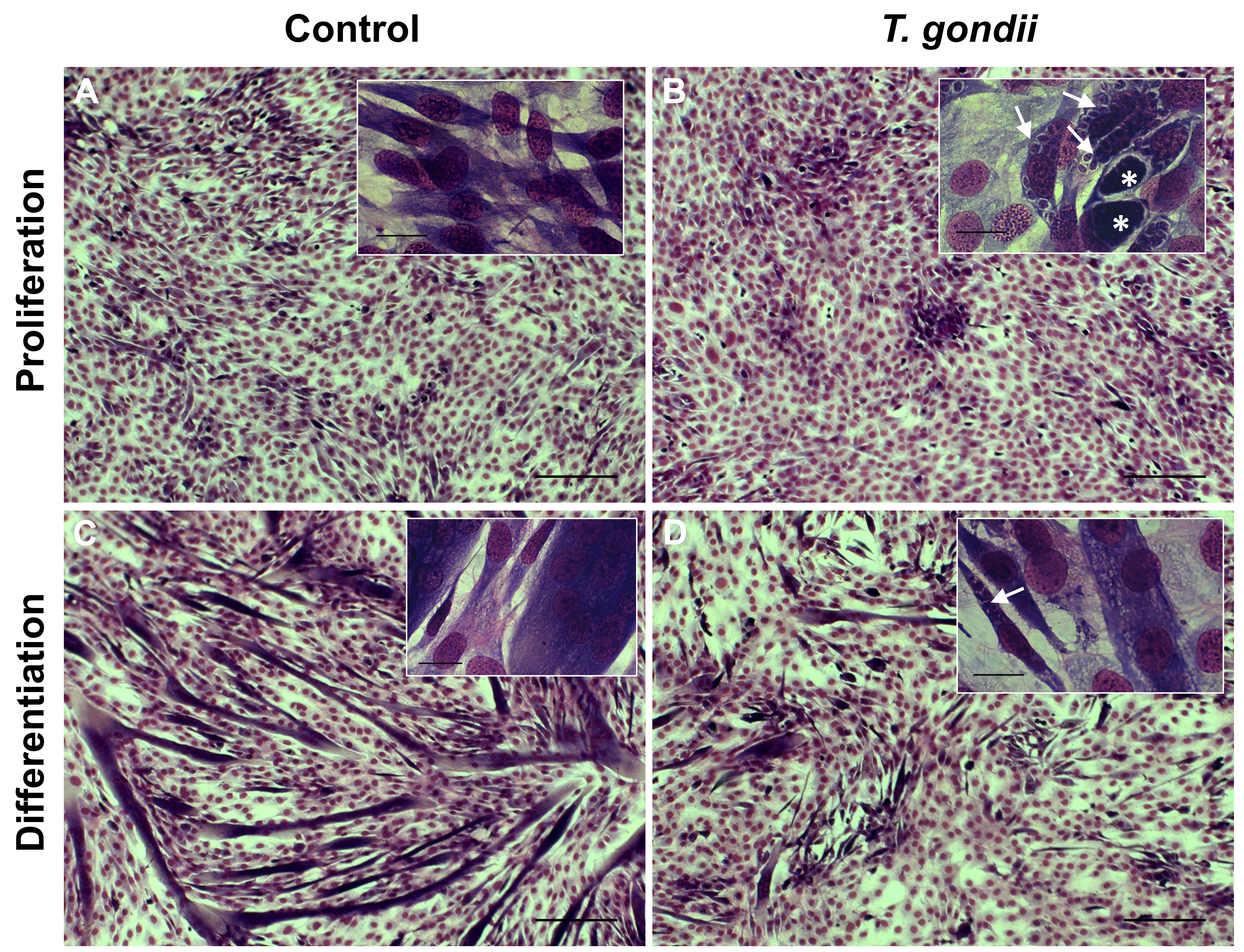

Supplement: Figure S2 — Culture evaluation with Giemsa stain after 120 h of induction of myogenesis. (A,B) PM-treated cells, (C,D) DM-treated cells. Infected cultures are on the right panels. Parasites within parasitophorous vacuoles are indicated by arrows and cyst-like structures are indicated by asterisks (*). Scale bars: 200 μm. Insets: 20 μm. [file Image_2.TIF]
